# Effect of addition of hydroxyapatite nanofibers to glass ionomer cement on fracture toughness, microhardness, and fluoride release

**DOI:** 10.1007/s10266-026-01308-4

**Published:** 2026-02-05

**Authors:** Mai Samy Sheta, El‑Refaie Kenawy, Samar A. Khattab

**Affiliations:** 1 Lecturer, Dental Biomaterials Department, Faculty of Dentistry, Tanta University, El-Giesh St., Tanta, Gharbia Egypt; 2 Chemistry Department, Faculty of Science, Tanta University, El-Giesh St., Tanta, Gharbia Egypt

**Keywords:** Glass ionomer cement, Hydroxyapatite nanofibers, Fracture toughness, Microhardness, Fluoride release

## Abstract

The current study attempted to investigate the effect of hydroxyapatite nanofibers (HANFs) added in different percentages upon the qualities of glass ionomer cement (GIC): fracture toughness, vickers microhardness and fluoride release. Synthesis and characterization of hydroxyapatite nanofibers were carried out before being added to the powder of high-viscosity conventional GIC in three varied weight ratios (1, 3 and 5%) resulting into 3 experimental groups which were next compared with basic GIC as a control group. Fracture toughness, Vickers microhardness and fluoride release on day1 and day 7 were evaluated. Results indicated significant improvement in fracture toughness, microhardness and fluoride release of GIC modified by hydroxyapatite nanofibers at varied tested ratios and this increase was directly proportional to their ratio of addition. According to these findings, glass ionomer cement modified with hydroxyapatite nanofibers in varying proportions could be a dependable restorative material with enhanced fracture toughness, microhardness and fluoride release.

## Introduction

Clinical dentistry has made considerable use of glass ionomer cement (GIC) since Wilson and Kent first introduced it in 1971.GIC, an acid–base cement, is made up of ion-leachable glass, polymeric acid, and water. All elements go through a setting reaction where the basic glass powder neutralizes the acid [1].

The special qualities of GIC, such as its high adherence to tooth structure, are the primary reason for its successful and efficient application in clinical dentistry [2]. The adhesion characteristic of GIC is used to fill up the small spaces at the tooth-restoration contact. Because of its low coefficient of thermal expansion, GIC is insensitive to changes in temperature [3]. GIC has fluoride ion release properties that prevent cavity advancement [4]. Since GIC doesn’t irritate the pulp or have any carcinogenic qualities, it is also very biocompatible with the tissues of teeth. Despite its widespread usage, GIC has numerous limitations such as low fracture toughness, which makes it susceptible to cracking [5]. GIC is a brittle material and cannot withstand severe stress, resulting in fast breakages [6]. GIC’s sensitivity to moisture and desiccation limits its application in high-stress situations [7].

Recent improvements are aimed at addressing some of these drawbacks. For example, the development of high-viscosity GIC with improved mechanical properties and wear resistance enabled filling of posterior teeth during atraumatic restorative treatment (ART) procedures [8]. Resin-modified glass ionomer cement was created with lower moisture sensitivity than conventional GIC [9]. However, the cytotoxicity and biological impacts of this kind of material are still undetermined [10, 11]. There is clearly a need for an esthetic restorative material that has high biological and mechanical qualities.

Hydroxyapatite [Ca_10_(PO_4_)_6_(OH)_2_] is a naturally existing bone mineral that has great biological properties. Its crystal structure and content are comparable to those of natural apatite, which is present in the skeletal system and hard dental tissues. Its applications include improving surface hardness, toughness, and biocompatibility [12]. Yap et al. [13] found that incorporating hydroxyapatite particles into GIC increased its hardness. The mechanical characteristics of GIC containing hydroxyapatite were investigated by Lucas et al. [14], who found that it improved fracture toughness and long-term bonding to dentin.

Recent research has shown that nano-HA crystals enhance enamel remineralization [15]. Nano-HA also has improved mechanical characteristics of GIC [16]. The addition of 10% nano-hydroxyapatite particles into GIC boosted fluoride ion release and recharging capacity, hence improving its anti-cariogenic capability [12].

Nanofibers remain an important subset of biomaterials due to their numerous biological applications. Electrospinning is a simple and successful method for creating continuous nanofibers from submicron to nanoscale dimensions by applying a high potential electric field. The procedure produces solid threads from solution without the use of coagulation chemicals or high temperatures [17–19]. Electrospun nanofibers provide several advantages over normal fibers, including larger surface areas. High porosity, small pore size and excellent fiber interconnectivity are other characteristics of electrospun nanofibers. Another benefit is that the composition of the nanofiber can be controlled to provide the desired functionality or attribute, giving surface functionalities more flexibility [20, 21].

In this study, hydroxyapatite nanofibers were prepared and characterized then added in different ratios to GIC. The hypothesis of this investigation was that addition of hydroxyapatite nanofibers (HANFs) would impact fracture toughness, microhardness and fluoride release of GIC.

## Materials and methods

### Materials

Polyvinyl alcohol (PVA) with an average molecular weight of 85,000–124,000 g/mol and an 89% degree of hydrolysis was provided by Sigma-Aldrich (USA). Polyvinyl pyrroliddone, (PVP) (average mol wt 40,000 g/mol) was purchased from techno pharmchem company (India). Calcium nitrate tetrahydrate (Ca(NO_3_)_2_.4H_2_O) was obtained by Morgan specialty chemicals (Egypt). Dipotassium hydrogen phosphate (K_2_HPO_4_) was purchased from Biochem company (Egypt). High-viscosity GIC (fuji IX, GC corporation, Tokyo, Japan) was utilized.

### Synthesis of hydroxyapatite nanofibers

#### Preparation of hydroxyapatite (HA)

Calcium nitrate tetrahydrate (Ca (NO_3_)_2_.4H_2_O) (1.18 g) and dipotassium hydrogen phosphate (K_2_HPO_4_) (0.86 g) were dissolved in 50.0 mL of distilled water to make 0.1 M solutions. Subsequently, 10.0 ml of 0.10 M dipotassium hydrogen phosphate and 16.7 ml of 0.10 M calcium nitrate tetrahydrate were added dropwise to create suspensions of Ca/P molar ratios at 1.67 and pH at 9. After the aging period, the precipitate was put in a 125 ml Teflon reactor, inserted into a stainless-steel autoclave, and held in an oven set at 200 °C for 24 h before naturally cooling to room temperature. The product was rinsed with water multiple times until the pH reached 7, yielding a fine white precipitate of HA particles. The precipitate was dried for 24 h at 150 °C [22].

### Preparation of hydroxyapatite/PVA/PVP solution

Aqueous PVA solution (8%) was made by dissolution of 0.8 g in 10 mL of distilled water, then stirred continuously at 90 °C till homogeneous (about 2 h). Then, PVP (0.4 g) was added to PVA solution. After obtaining clear solution, 0.05 g of hydroxyapatite was blended with the polymer solution by stirring for 24 h at ambient temperature to ensure good dispersion.

### Fabrication of HA/PVA/PVP composite nanofibers by electrospinning

HA/PVA/PVP composite nanofibers were prepared by injecting the polymer solution into the electrospinning device (model E02S, NANOFIBER LABS China). The HA/PVA/PVP solution was inserted into a syringe, and electrospun at a flow rate of 0.5 mL/h under high-voltage power source (10 kV). The nanofibers were collected on grounded aluminum foil.

### Obtaining pure HA nanofibers

To produce pure HA nanofibers, electrospun HA/PVA/PVP composite nanofibers were heated to 350 °C for 2 h at a rate of 2 °C/min, then calcinated for 1 h at 600 °C [23].

### Characterization techniques

The structural morphology of HA-incorporated polymer nanofibers was observed by a field emission-scanning electronic microscope SEM (JEOL JSM 6510 lv, Japan). Prior to SEM analysis, all the specimens were coated with gold by low-vacuum sputter coating. Fourier transform infrared spectroscopy FTIR spectral analyzed data a ratio recording Spectrophotometer (Perkin-Elmer, model 1430) using KBr disk pellets with wavelength numbers ranging from 4000 to 500 cm^−1^. A Rigaku *X* ray diffractometer (Rigaku, Tokyo, Japan) was used to measure the *X* ray diffraction (XRD) with Cu K (= 1.5418 Å) and 2̟ from 5 to 60 at a scanning rate of 0.02°/min. FTIR and XRD were measured before and after calcination to measure structural make-up, content and crystallinity of structures and check out formation of pure HA nanofibers. Thermogravimetric Analyzer (TGA 4000, Perkin Elmer Instruments) was used to perform thermogravimetric analysis (TGA) for assurance of thermal decomposition of polymer while thermal stability of HA after calcination step; specimens weighing 5–10 mg were scanned at 50 °C to 800 °C at a rate of 30 °C/min surrounded by a nitrogen environment.

### Sample preparation and grouping

HANFs were added to GIC powder in percentages of (0 control and 1, 3 and 5% for experimental groups by the weight of the powder) by an electronic balance (Radwag Wagi Elektronic, AS 220.B,509,892, Poland) at an accuracy of 0.0001 g utilizing the geometric dilution approach prior to mixing with the liquid. The studied groups were control group which had 0% HANFs and experimental groups: 1% HANFs, 3% HANFs and 5% HANFs. These concentrations of added HANFs were chosen to explore the effect of low, moderate, and relatively high HA content on the mechanical properties and fluoride release of GIC. Concentrations below 1% were expected to have minimal effect, while concentrations above 5% could compromise the handling, mixing, and homogeneity of the cement. This range allows a systematic evaluation of the material’s performance while maintaining clinical applicability. 12 specimens were made for every property for every group (*n* = 12). Based on a prior study, the sample size for this investigation was established [14]. It was estimated using a computer application G power version 3.1.9. The significance threshold was 0.05 and the sample size’s power was over 80% for this investigation with a confidence range of 95%. The effect size is 1.6996732. The specified ratio of 3.6 powder/1 liquid of high viscosity GIC was used to prepare each specimen at room temperature. Specimens were manually mixed by a plastic spatula over a glass slab. For every test, a Teflon mold with the required measurements was made. The mold was filled with the mixture. It took 2 min and 30 s to set. By applying physical pressure to both sides of the specimen, glass microscopic slides with transparent polystyrene matrix sheets were used for extruding excess material. After an hour, the specimens were taken out of their mold and ground using 600-grit wet silicon carbide paper. Prior to fracture toughness and microhardness evaluation, incubation of the specimens was done in distilled water for 24 h (BTC, BT1120, Biotech, Cairo, Egypt). The design and procedures of the present study were accomplished according to the research guidelines adopted by the research ethics committee, faculty of dentistry, Tanta University (#R-BIO-12–24-3166).

## Testing properties

### Fracture toughness

**Fig. 1:**
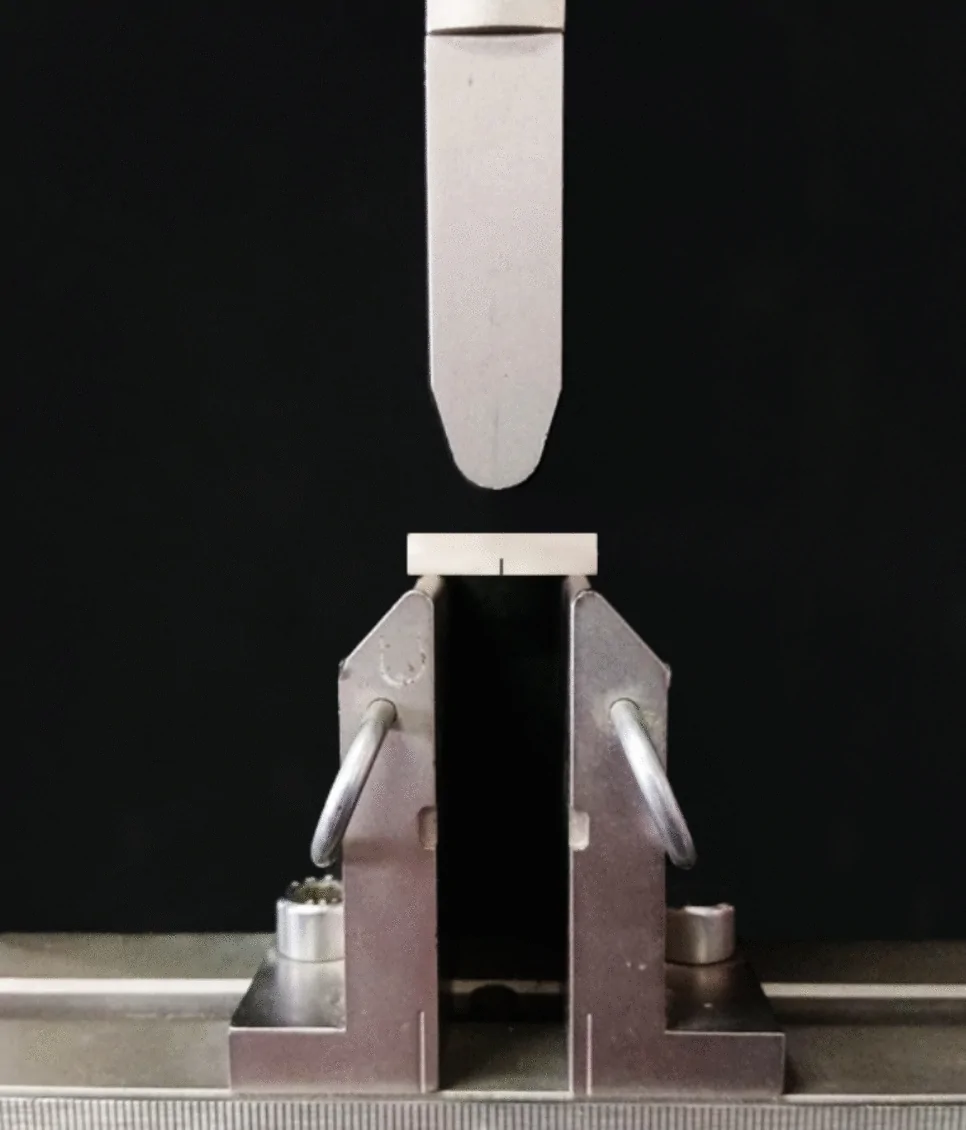
Fracture toughness testing of the specimen

Single-edge-notched-beam specimens of 25 mm length, 5 mm width and 2.5 thickness were prepared according to adapted American society for the testing of materials (ASTM) E-399 standard methods to determine the fracture toughness [24]. A precisely sized groove that was made in middle of the teflon mold, allowing the notch to be located in the center. A sharp central crack was made in the specimen by inserting a steel blade into the prepared groove until it reached the mid-width of the mold before the cement set. Using a universal testing machine (Instron, Model: 3365, Norwood, USA), the specimens were tested in 3 point bending mode by placing them on 2 supporting wedges (4 mm in diameter) spaced 20 mm apart, with the notched side facing downward. The loading wedge (10 mm in diameter) applied load at the center of the specimen on the opposite side of the notch. At a crosshead speed of 1.0 mm/min **(**Fig. 1). Calculation of fracture toughness K_Ic_ was done by Eq. (1), provided by ISO 12737:2010 [25] and result was in (MPa m.^1/2^)

1 $$K_{Ic} = \, \left( {P/B \, x \, L/w^{1.5} } \right) \, x \, f \, (a/w)$$

where: P = Maximum Load, B = Specimen thickness, L = Supporting span, w = Specimen width, a = notch length and f(a/w) = a geometrical function of a and w that was calculated using Eq. (2), obtained from ISO12737:2010 [25].

2 $$F \, \left( {a/W} \right) \, = \, 3 \, \left( {a/W} \right)^{1/2} \left[ {1.99 \, - \, \left( {a/W} \right) \, \left( {1 \, - \, a/W} \right) \, \left\{ {(2.15 \, - \, 3.93 \, \left( {a/W} \right) \, + \, 2.7 \, \left( {a/W} \right)^{2} } \right\}} \right] \, / \, 2 \, \left( {1 \, + \, 2a/W} \right) \, \left( {1 \, - \, a/W} \right)^{3/2}$$

### Vickers microhardness

Cylindrical specimens with 3 mm thickness and 6 mm diameter were made. Following setting, finishing of the specimens was made by wet 800, 1000, and 1200-grit silicon carbide paper. A digital microhardness tester (Zwick/Roell, INDENTEC, ZHVµ-S, West Midlands, England) was used to conduct Vickers microhardness testing. A 50 gf load was delivered by the indenter over a 30 s dwell period [26]. Three measurements were taken of each specimen, and the average was determined. The Vickers hardness number (VHN) was calculated by the device’s software.

### Fluoride release

Specimens of 2 mm height and10 mm diameter were prepared [14]. Then, every single specimen was placed in 5 ml of deionized water in a firmly sealed glass bottle at 37 °C in incubator (BTC, BT1120, Biotech, Cairo, Egypt). After 24 h, bottles were carefully shaken and the specimens were withdrawn, rinsed by 1 ml of deionized water then dried and reimmersed into another bottle with 5 ml of fresh deionized water that was replaced every 24 h. For 7 days, each specimen underwent the same procedure as before. To prevent the development of fluoride complexes, 0.5 ml of total ionic strength adjustment buffer (TISAB III) was added to the obtained solution [27]. The ion-selective electrode technique [28] was used to measure fluoride ion concentration (μg/mL) in the deionized water on day1 and day 7. To estimate the total fluoride in μg, the μg/ml values were multiplied by the amount of the tested solution. The fluoride release in μg/cm^2^ was calculated by dividing the total fluoride by the specimen’s area.

### Statistical analysis

The statistical package for social sciences (IBM SPSS Statistics version 26) was used to collect and analyze the data. Means, standard deviations, and ranges were used to display numerical variables. One Way ANOVA test was employed for the intergroup comparison, and a *P* value** < **0.05 (*) denoted a significant difference, while a *P* value** < **0.001 (**) denoted a highly significant difference. Then, Tukey multiple comparison test was applied for pairwise analysis to assess variation between each pair of groups.

## Results

### Characterization results

**Fig. 2:**
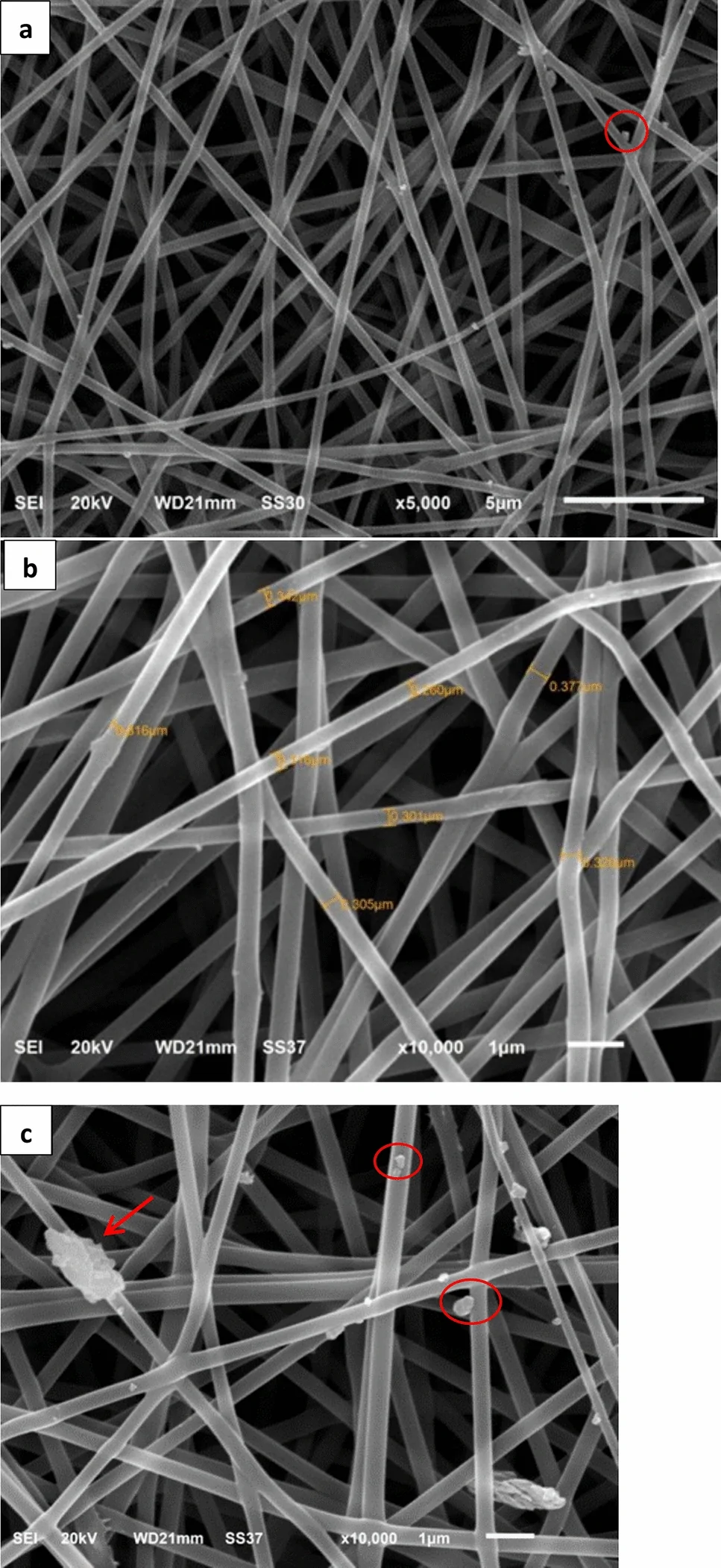
SEM images of HA-incorporated (PVA/PVP) nanofibers: **a** at 5 × and **b**, **c** at 10 × magnifications

The morphological structure of the HA-incorporated (PVA/PVP) nanofibers is shown in (Fig. 2). SEM images displayed uniform and smooth surfaces of the polymer composite nanofiber (HA/PVA/PVP); their diameter ranged from 260 to 342 nm. Some aggregates of hydroxyapatite particles were formed on the nanofiber surface, which proved that HA was successfully incorporated along the polyvinyl alcohol/polypyrrolidone fibers.

**Fig. 3:**
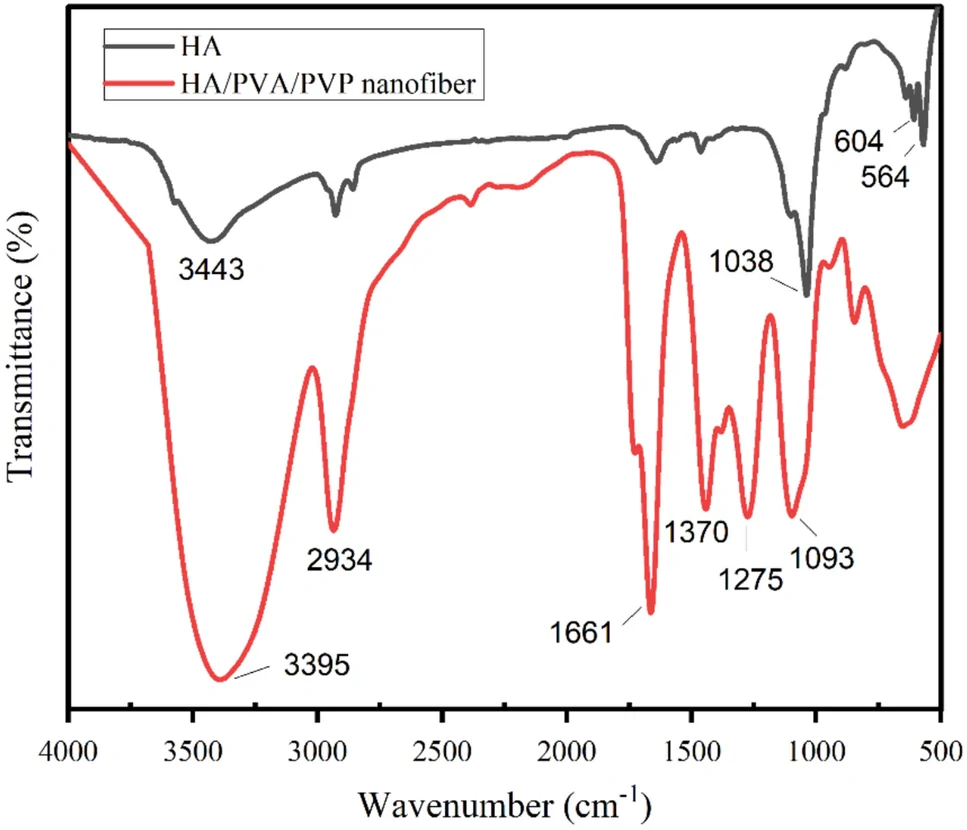
FTIR spectra of HA and HA-incorporated (PVA/PVP) nanofiber

The FTIR spectra of the hydroxyapatite (HA) and the polymer composite nanofiber are shown in Fig. 3. HA exhibited characteristic peaks at 3573 and 3443 cm^−1^, attributed to stretching vibration of hydroxy group (OH). Peak at 1038 cm^−1^, was assigned for the P − O stretching vibration, and at 604 and 562 cm^−1^ was assigned for the O − P − O bending vibration of [29, 30]. The FTIR spectrum of the composite nanofiber displayed peak at 3395 cm^−1^ which, was attributed to the stretching vibration of hydroxyl group (OH) of polyvinyl alcohol in the polymer blend. The stretching vibrations of C-H appeared at 2934 cm^−1^.The absorption bands located at 1661, 1370, 1275 cm^−1^, attributed to the C = O stretching of, lactone structure and bending vibrations of the C–N present in polypyrrolidone, respectively [31, 32].Overall, the presence of hydroxyapatite functional groups such as PO_4_^3−^, OH − , proved the deposition of HA on polymer blend (PVA/PVP).

**Fig. 4:**
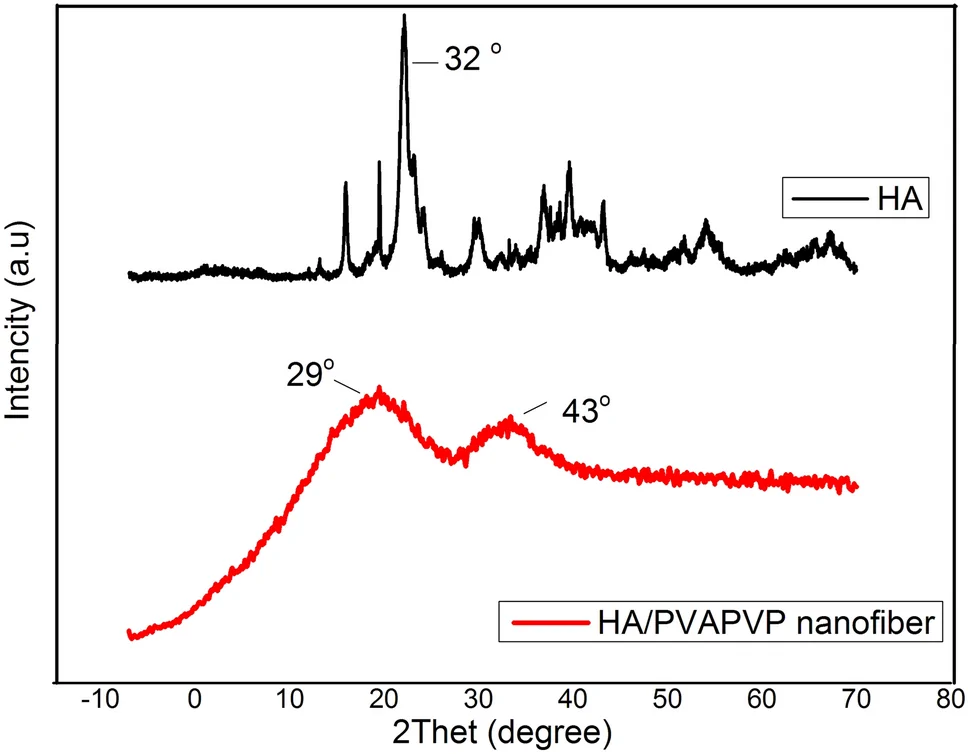
XRD pattern of HA and HA-incorporated (PVA/PVP) nanofiber

The XRD data of HA/PVA/PVP composite nanofiber (Fig. 4) exhibited 2 broad diffraction peaks (halos) at 2θ = 29° and 43° which, corresponds to the diffraction peaks of PVA and PVP blend [33]. There was a peak with low intensity appeared at 2θ = 39.5° which, proved the incorporation of HA onto polymer nanofibers. HA nanofibers showed a peak at 2θ = 32°, which corresponds to the main peak of the hexagonal HA structure [34].

**Fig. 5:**
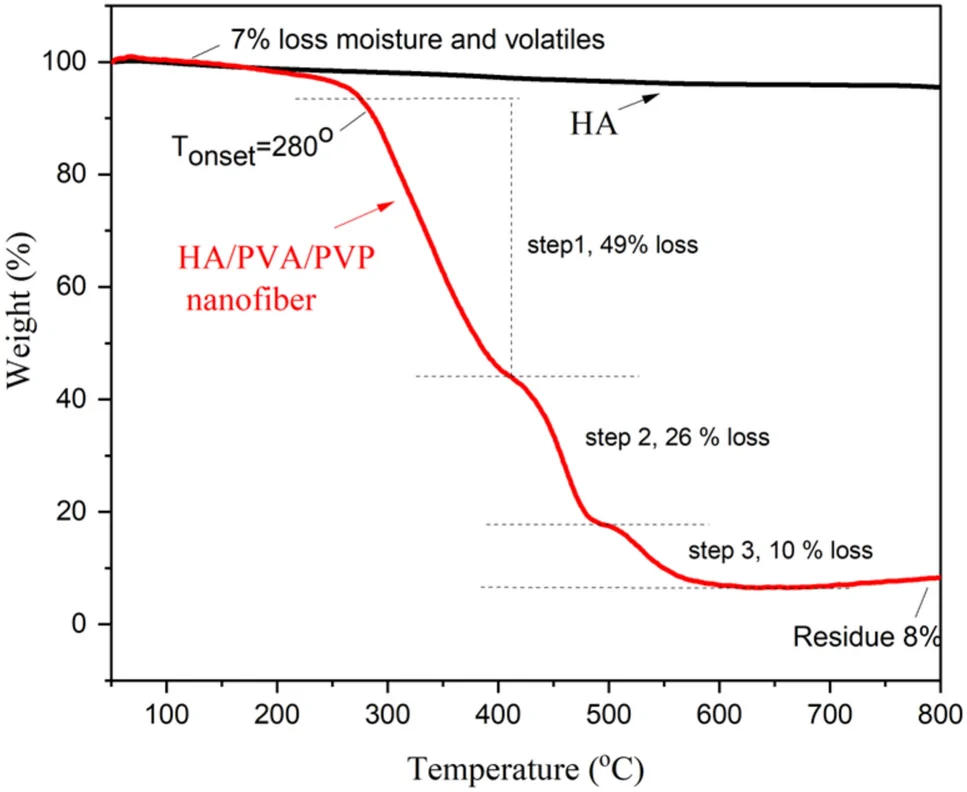
TGA curves of HA and HA-incorporated (PVA/PVP) nanofiber

The thermal decompositions of Hydroxyapatite (HA) and HA-incorporated (PVA/PVP) nanofiber were assessed by TGA analysis as shown in Fig. 5. HA demonstrated good thermal stability over the whole temperature range with a slight weight loss of about 5% up to 800 °C, this agreed with a prior study recorded by Becerra et al. [30].

On the other hand, TGA curve of the composite nanofiber (HA/PVA/PVP) displayed 3 steps of weight loss. In the beginning, there was a weight loss of 7% due to release of moisture from the hydrophilic polymers. The HA-incorporated (PVA/PVP) nanofiber started to decompose at 280 °C losing about 49% of its weight to 418 °C which, could be caused by the degradation of polymer chains [35]. The second weight loss occurred within the range of 418–500 °C, losing 26%. The third step of degradation occurred within the temperature range of 500–580 °C losing 10% of its weight. The remaining residual weight was 7% at 800 °C.

## Testing results

Means and standard deviations for the values of fracture toughness, vickers microhardness and fluoride release of the tested groups are listed in Table 1

**Table 1:** One-way ANOVA results of fracture toughness, vickers microhardness and fluoride release for studied groups

Groups	Fracture toughness MPam^1/2^	Vickers microhardness (VHN)	Fluoride release (µg/cm^2^)	
			Day 1	Day 7
control	0.47 ± 0.02^A^	36.08 ± 1.084^A^	41.74 ± 1.63^A^	26.29 ± 2.73^A^
1% HANFs	0.57 ± 0.016^B^	42.46 ± 0.73^B^	46.45 ± 2.46^A^	33.12 ± 2.59^B^
3% HANFs	0.72 ± 0.02^C^	49.80 ± 0.56^C^	64.58 ± 4.67^B^	45.05 ± 3.28^C^
5% HANFs	1.08 ± 0.08^D^	61.60 ± 1.24^D^	133.0 ± 9.37^C^	74.18 ± 4.82^D^
*P* value	0.0001**	0.0001**	0.0001**	0.0001**

P: ANOVA test, significance: p*<0.05, p**<0.001

Common capital letter in the same column indicates non−significant difference, different capital letter in the same column indicates significant difference

ns not significance

### Fracture toughness

One-way ANOVA showed a statistically high significant difference between all groups (*p* < 0.001). Pair wise analysis using Tukey’s test revealed the control group recorded highly significantly lower fracture toughness than all experimental groups which were highly significant different from each other (*p* < 0.001). The increase in fracture toughness values was directly proportional to HANFs percentages of the experimental groups. 5% HANFs group was markedly significantly higher than other tested groups (*p* < 0.001).

### Vickers microhardness

One-way ANOVA showed that all groups were highly significant different from each other (*p* < 0.001). Pair wise analysis using Tukey’s test revealed that the vickers microhardness of control group was significantly lower than the experimental groups (*p* < 0.001). Vickers microhardness values were increased progressively with HANFs percentages of the experimental group reaching peek at 5%HANFs group.

### Fluoride release

Data from Table 1 indicated that control and experimental groups recorded a decline in fluoride ion release on day 7 in comparison to day 1. On day 1, no significant difference was recorded between fluoride release values of control group and 1% HANFs group and they were highly significantly lower than other tested groups. 5% HANFs group was markedly significantly higher than group 3% HANFs group (*p* < 0.001). While on day 7, control group was highly significantly lower than other experimental groups which their fluoride release values were progressively increased significantly with HANFs ratios as the highest value recorded by 5% HANFs group (*p* < 0.001).

## Discussion

Glass-ionomer cements (GICs) are frequently employed in numerous fields of dentistry. Among the advantages of GIC, it may be inserted in cavities without the requirement for bonding steps because of their high adhesion to tooth structures [36]. Biocompatibility was originally reported in GICs, which had a linear coefficient of thermal expansion like dentin. Another feature of their smart behavior is the capacity to release and recharge fluoride [37]**.**

Electrospinning is a simple and adaptable way of producing very thin fibers from a range of components such as polymers, ceramics and composites. Nanofibers have unique properties, including high surface area per unit mass (e.g., nanofibers with a diameter of about 100 nm have an average surface area of about 1000 m_2_/g), high porosity, exceptional mechanical qualities coupled with remarkable flexibility and the ability to control the chemical makeup of the nanofibers with an extensive selection of their qualities. A range of nanoparticles or nanofillers can be joined using the electrospinning technique and subsequently encapsulated in a nanofiber matrix. Electrospun composite nanofibers can be generated by distributing the nanoparticles in the polymer solutions [38].

Hydroxyapatite (HA) was prepared by a hydrothermal method as high crystallinity and relative stoichiometry are characteristics of the hydroxyapatite powders generated by this method [39, 40]. The structures of HA and HA/PVA/PVP composite nanofiber were investigated by several techniques such as FTIR spectroscopy, XRD, SEM, besides the thermal stability by TGA**.**

Fracture toughness is a term that describes the material’s capacity to withstand catastrophic failure and is more accurate determinant of clinical performance of GIC than compressive or diametral strength measurements [14, 41]. Fracture toughness is defined as the instantaneous stress situation (K_C_ value) at the onset of crack propagation [42, 43]. The essential metric known as KIC (plane-strain fracture toughness) represents the low limiting value of KC in the tensile opening mode (mode I), which is also considered to be a useful indicator of marginal fracture and wear resistance, both of which are important for restorative materials [44]. Regretfully, none of the existing GIC testing standards include any specification for this feature. The single-edge notch test, which is based on ASTM specification E-399 [24], which was employed in this investigation for testing fracture toughness, was used by several investigators for testing composites and cements, including glass ionomers [14, 45–47]. One crucial characteristic that is connected to a material’s resistance to penetration and indentation is microhardness [48]. In addition to serving as indications of wear resistance of the materials, surface hardness tests can be used to evaluate the durability and deterioration of dental materials [49]. The most precise, easily available, and straightforward test for determining the microhardness of brittle materials like glass ionomer cement, is the vickers microhardness test [50].

In this study, addition of HANFs has markedly improved fracture toughness and microhardness of GIC and this improvement was directly proportional to HANFs ratio with the highest value recorded by 5% HANFs. These findings were consistent with Lucas et al. [14], who reported that adding hydroxyapatite to GIC resulted in significantly greater fracture toughness after 15 min and 24 h of mixing. Barandehfard et al. [51] measured the microhardness of GIC containing nanohydroxyapatite by storage in distilled water at 37 °C for 1 and 7 days and found that after 7 days, the microhardness of GIC modified by nanohydroxyapatite was double that of GIC alone. Additionally, Al-Hamaoy et al. [52] found that, in contrast to microhydroxyapatite, the addition of nanohydroxyapatite improved the microhardness of GIC. Hydroxyapetite nanofibers’ structure have contributed to improved mechanical performance as these electrospun nanofibers possess high porosity and high fiber interconnectivity. Their high surface to volume ratio increased the contact area between the nanofibers and the matrix which resulted into better mechanical interlocking between them that ensured transferring the load to the stronger nanofibers and promoted toughening mechanisms due to improving the behavior of each nanofiber as a crack stopper so increasing the material’s resistance to crack propagation [53].

GIC’s ability to release fluoride ions is essential for lowering the incidence of dental caries by integrating into tooth tissue to produce fluoroapatite, decreasing enamel and dentin solubility, and inhibiting growth of the bacteria [54, 55]. The specimens in this investigation were stored in deionized water, which serves as a baseline for fluoride release potential in unstimulated settings. [27, 56, 57]. According to the study’s findings, all examined groups had a decline in fluoride release on day 7 after a higher level on day 1. These findings were consistent with various research and are referred to as “the initial burst effect” [27, 58, 59]. This is a beneficial effect because it encourages enamel/dentin remineralization and decreases the survival of germs remaining in the cavity [60]. During the setting reaction, the glass particles dissolve in polyacrylic acid, releasing the first burst of fluoride. Furthermore, fluoride diffuses rapidly from the matrix exposed on the specimen’s surface, which could explain the high levels of fluoride release on day 1. In contrast, fluoride’s ability to diffuse through cement pores accounts for the continuous fluoride release during the following days [61]. In this study, nanofibers addition to glass ionomer has improved its fluoride release on different period measurement and it was proportional to the ratio of nanofibers. This is owing to high porosity of the nanofibers, also due to the greater surface area of hydroxyapatite nanofibers, which resulted to increased acid–base reactivity, thereby enhancing fluoride ion release.

These improvements caused by nanofibers addition are in consistent with Nishimura et al. [62] who tested how adding powdery cellulose nanofibers (CNF) affected the glass ionomer cement’s characteristics. It was discovered that the quantity of fluoride ions released increased as the CNF loading increased. Also, Alatawi et al. [63] reported that in comparison to conventional GIC, the GIC containing nanohydroxyapatite showed increased fluoride ion release. According to a different study by Moheet et al. [64], GIC treated with nanohydroxyapatite-silica released more fluoride ions than conventional GIC. According to Moshaverinia et al. [16], hydroxyapatite added GIC showed a greater release of fluoride than conventional GIC. The hypothesis of this study was accepted as HANFs addition to GIC has significantly improved fracture toughness, microhardness and fluoride release.

Limitations of this study include that only one commercial type of conventional GIC was tested. No clinical simulation of thermal conditions and occlusal forces by thermomechanical aging. Higher percentages of HANFs should be investigated. Fluoride ion release on longer periods should be assessed.

## Conclusions

The following conclusions could be drawn within the constraints of this study: The incorporation of HANFs into to glass ionomer cement has demonstrated enhanced fracture toughness, microhardness and fluoride release. These enhancements were positively correlated with the concentration of HANFs, indicating a dose-dependent effect.

## Author’ contributions

The manuscript was reviewed by all authors. A. Mai Samy Sheta: (authored and carefully revised the study, and helped with its conception, design, and interpretation.). B. El‑Refaie Kenawy: (helped with its idea, contributed to the acquisition, interpretation, and critical revision of the work.). C. Samar A. Khattab: (Reviewed the manuscript critically and helped with its conception, design, and interpretation.)

## Data Availability

All data are available from the corresponding author upon reasonable request.
